# Selenium nanoparticles alleviate ischemia reperfusion injury-induced acute kidney injury by modulating GPx-1/NLRP3/Caspase-1 pathway

**DOI:** 10.7150/thno.70830

**Published:** 2022-05-09

**Authors:** Shaobo Wang, Yin Chen, Songling Han, Yong Liu, Jining Gao, Yinghui Huang, Wei Sun, Junping Wang, Cheng Wang, Jinghong Zhao

**Affiliations:** 1 Department of Nephrology, The Key Laboratory for The Prevention and Treatment of Chronic Kidney Disease of Chongqing, Xinqiao Hospital, Army Medical University (Third Military Medical University), Chongqing, 400037, China.; 2 State Key Laboratory of Trauma, Burns and Combined Injury, Institute of Combined Injury of PLA, Chongqing Engineering Research Center for Nanomedicine, College of Preventive Medicine, Army Medical University (Third Military Medical University), Chongqing, 400038, China.; 3 Biomedical Analysis Center, Army Medical University (Third Military Medical University), Chongqing, 400038, China.

**Keywords:** acute kidney injury, ischemia reperfusion injury, selenium nanoparticles, glutathione peroxidase-1, inflammasome

## Abstract

**Rationale:** Acute kidney injury (AKI) is a common critical illness in the clinic and currently lacks effective treatment options. Ischemia reperfusion injury (IRI) is a major pathogenic factor for AKI. Due to the deficiency of selenium (Se) in AKI patients, we intended to treat IRI-induced AKI using a Se rebalancing strategy in the present study.

**Methods:** Sodium selenate, ascorbic acid, and bovine serum albumin (BSA) were employed to prepare nanomaterials termed Se@BSA nanoparticles (NPs) using a simple method. Experiments with human renal tubular epithelial HK-2 cells exposed to hypoxia/reoxygenation (H/R) and IRI-AKI mice were used to evaluate the therapeutic efficiency of Se@BSA NPs. Transcriptome sequencing, further molecular biology experiments, and pathologic analysis were performed to investigate the underlying mechanisms.

**Results:** Se@BSA NPs accumulated in mouse kidneys and could be endocytosed by renal tubular epithelial cells after intravenous administration. *In vitro* studies showed that Se@BSA NP treatment markedly increased the levels of glutathione peroxidase (GPx)-1 and suppressed NLRP3 inflammasome activation in H/R cells, which resulted in reductions in the proteolytic cleavage of pro-Caspase-1 into active Caspase-1 and the maturation of inflammatory factors. Mouse experiments confirmed these findings and demonstrated an inspiring mitigative effect of Se@BSA NPs on IRI-induced AKI. Owing to modulation of the GPx-1/NLRP3/Caspase-1 pathway, Se@BSA NPs dramatically inhibited fibrosis formation after AKI.

**Conclusion:** This study provides an effective therapeutic option by applying easy-to-produce Se-containing nanomaterials to remedy Se imbalance and impede inflammatory responses in the kidney, which is a promising candidate for AKI treatment.

## Introduction

Acute kidney injury (AKI) is an urgent and severe disease observed in the clinic with high incidence and mortality, particularly for patients in the intensive care unit 1. Ischemia reperfusion injury (IRI), which causes renal injuries by inducing microvascular dysfunctions, mitochondrial damage, and the production of large amounts of reactive oxygen species (ROS) and proinflammatory cytokines 2,3, is one of the major etiological factors for AKI. The renal function of AKI patients rapidly deteriorates, manifesting as a reduction in the glomerular filtration rate and in the fluid and electrolyte imbalance and the accumulation of metabolic waste 4. Despite its limited effectiveness, the current treatment option for AKI is renal replacement therapy 5, which necessitates the development of effective alternative schemes.

The human body is a highly complex and balanced system, and tip the balance may result in illness, as exemplified by the occurrence of hypovitaminosis and iron-deficiency anemia 6,7. Encouragingly, rebalancing nutrient and molecular homeostasis is a useful therapeutic strategy. For instance, as AKI patients have a significant reduction in the levels of anti-inflammatory protein Klotho 8, which is abundant in the kidney, Klotho supplementation is instrumental in ameliorating AKI 9. Selenium (Se) is an essential trace element abundant in the kidney and is incorporated into selenoproteins, including glutathione peroxidases (GPxs). GPx-1 is known to be primarily expressed in healthy kidneys and accounts for almost all (> 96%) renal GPx activity 10. Previous studies showed that GPx-1 acted as an endogenous renoprotective agent but its expression was decreased after Se deficiency 11, which aggravates mesangial matrix expansion and pro-inflammatory responses 12. Considering the deficiency of Se in AKI patients 13, we proposed a treatment option by Se supplementation.

Selenium is instrumental for the prevention of diseases including cardiovascular disease, arthritis, cystic fibrosis 14, and cancers 15. Despite its wide use in dietary supplements, Se has a narrow therapeutic window with delicate toxicity margins 16. Selenium nanoparticles (SeNPs) have higher biocompatibilities than those of the inorganic and organic forms of Se and thus are alternatives for nutritional supplementation 17. Due to the important role of Se in stabilizing the immune system and activating host defense responses, SeNPs have advantages over other nanomaterials 18. There are increasing data demonstrating that SeNPs hold inspiring antimicrobial 19, antitumor 20,21, and antidiabetic properties 22, making the nanomaterial a hotspot for development into therapeutics 23. Additionally, SeNPs exhibit good prospects for their application in rapid diagnosis methods and even in the current COVID-19 pandemic 24.

Because the zero valence state of Se (Se^0^) exhibits lower toxicity than that of selenate (Se^II^), selenite (Se^IV^), or selenate (Se^VI^) ions 25,26, Se^0^ in the nano form is a promising candidate for biomedical applications. Generally, Se^0^-containing nanomaterials can be prepared using physical, chemical, and biological techniques 27. To simplify the preparation process, we fabricated a nanomaterial termed Se@BSA NPs using one-step chemical synthesis 28. Cell and mouse experiments were employed to evaluate the therapeutic effect of Se@BSA NPs on IRI-AKI. The underlying mechanisms were investigated by RNA-seq, other molecular biology experiments, and pathologic analysis. Se@BSA NPs act as biocompatible Se supplements to increase GPx-1 levels in injured kidneys, resulting in suppressions of NLRP3 inflammasome activation and the maturation of inflammatory factors. Accordingly, the easy-to-produce Se-containing nanomaterials are promising drug candidates for IRI-AKI treatment.

## Results and Discussion

### Preparation and characterization of Se@BSA NPs

**Figure 1:**
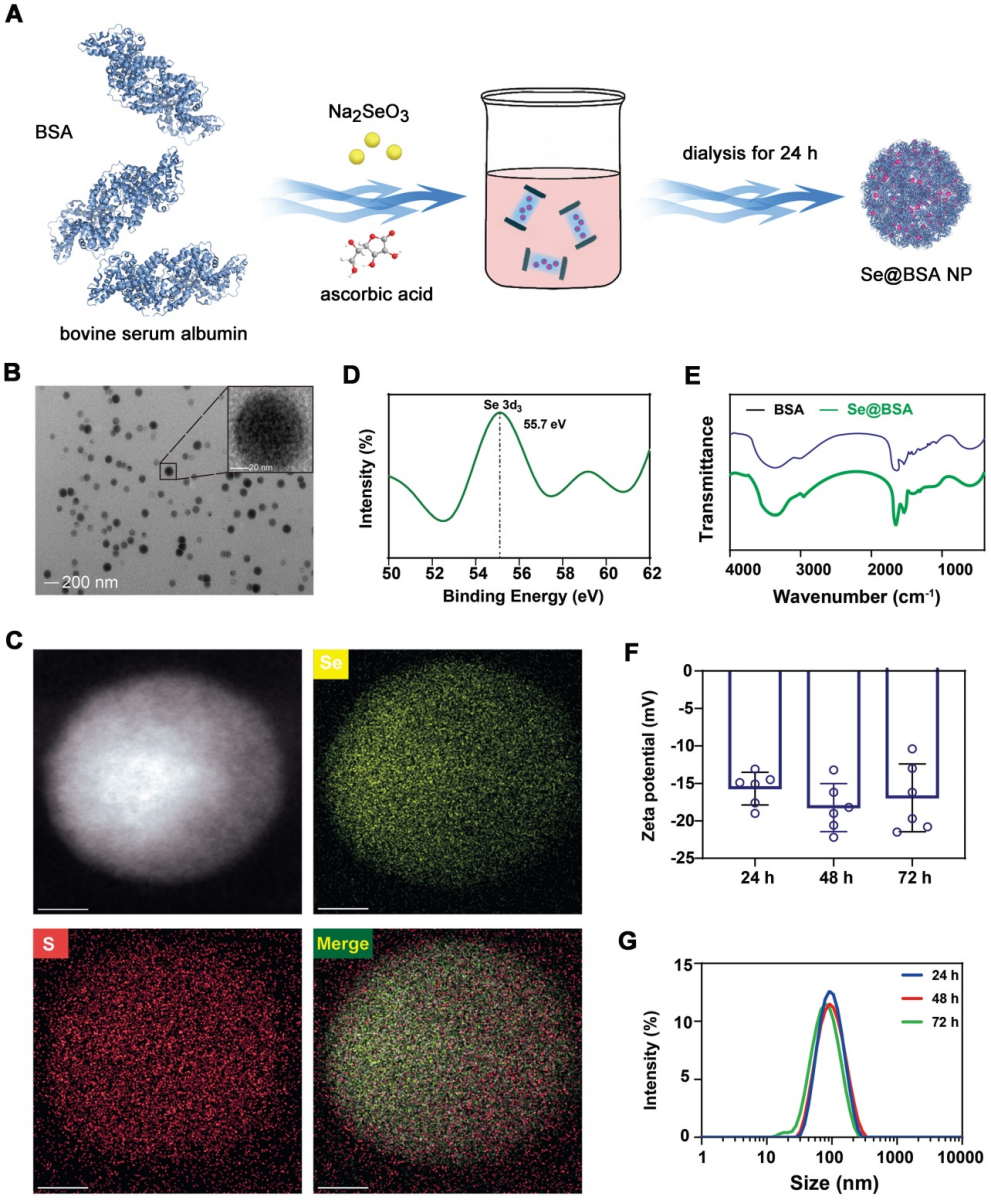
** (A)** Diagram depicting the preparation of Se@BSA NPs. **(B)** TEM image of Se@BSA NPs. The scale bar indicates 200 nm. The region of interest is magnified in the inset graph, in which the scale bare indicates 20 nm. **(C)** EDS images of Se@BSA NPs. The yellow and blue signals indicate Se and sulfur, respectively. The scale is 20 nm.** (D)** XPS spectrum of Se@BSA NPs.** (E)** FT-IR spectra of Se@BSA NPs. **(F)** Zeta potential determination. The results are presented as the mean ± SD. **(G)** DLS detection of the hydrodynamic diameters of Se@BSA NPs at different times.

Sodium selenate was reduced by ascorbic acid at a molar ratio of 1:2 29. To decrease the aggregation of the products, BSA was added for stabilization due to its biocompatibility and ease of acquisition (Figure 1A). The prepared compounds collected by dialysis were spherical particles with a preferable dispersity in solution and had an approximate size of 80 nm (Figure 1B). Energy dispersive X-ray spectroscopy (EDS) confirmed the presence of Se and S elements (Figure 1C). The Se content in the NPs was 17.7 μg/mg, as measured by inductively coupled plasma-mass spectrometry (ICP-MS). X-ray photoelectron spectroscopy (XPS, Figure 1D) indicated that Se presented as the zero valence state in the NPs 30. Fourier transform-infrared spectrometry (FT-IR, Figure 1E) and sodium dodecyl sulfate-polyacrylamide gel electrophoresis (SDS-PAGE, Figure S1) further revealed the existence of BSA. The average diameter of the negatively charged nanomaterial (-16.57 ± 1.27 mV, Figure 1F) termed Se@BSA NP was 88.4 nm, as determined by dynamic light scattering (DLS) (Figure 1G, polydispersity index: 0.25). Different batches of the products prepared on different days had comparable ζ-potentials and diameters (Figure S2). Se@BSA NPs were stable in PBS (pH: 7.4, Figure 1F-G), which laid a good foundation for the subsequent functional evaluation.

### Biocompatibility and biodistribution of Se@BSA NPs

**Figure 2:**
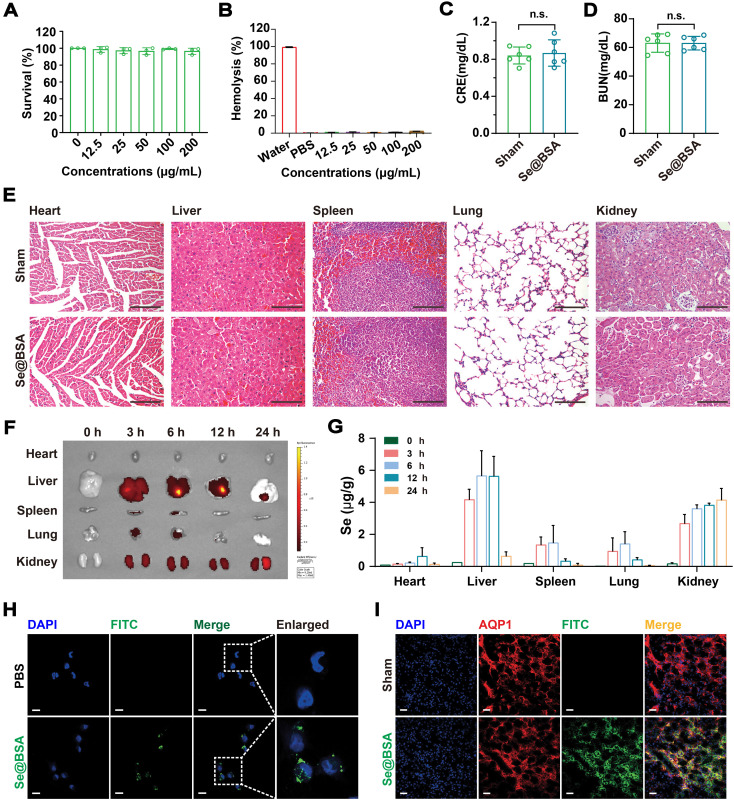
** (A)** Survival of HK-2 cells exposed to Se@BSA NPs. The results are shown as the mean ± SD. **(B)** Hemolysis of Se@BSA NPs. The results are shown as the mean ± SD.** (C)** Determination of CRE and BUN **(D)** contents in mice. The results are shown as the mean ± SD. n.s., not significantly.** (E)** HE staining of the organs of mice treated with sterile PBS and Se@BSA NPs. The scale bar indicates 200 µm. **(F)** *In vitro* imaging of FITC-labeled Se@BSA NPs in mouse hearts, livers, spleens, lungs, and kidneys at 0, 3, 6, 12 and 24 h post intravenous administration.** (G)** ICP-MS determining the content of Se element in mouse organs at different times after Se@BSA NP treatment.** (H)** Confocal images of HK-2 cells exposed to FITC-labeled Se@BSA NPs for 6 h. The scale bar indicates 20 µm. **(I)** Immunofluorescence microscopy revealing the colocalization of AQP1 (red) and FITC-labeled Se@BSA NPs (green) in mouse kidneys. The scale bar indicates 20 µm.

Se@BSA NPs were nontoxic to human renal tubular epithelial HK-2 cells (Figure 2A) and did not induce hemolysis at doses up to 200 μg/mL (Figure 2B, based on the weight of NPs). Intravenous injection (I.V.) of 20 mg/kg Se@BSA NPs was innocuous to C57 mice. There were no significant differences in body weight (Figure S3), serum alanine aminotransferase (ALT) and aspartate aminotransferase (AST) contents (Figure S4), or creatinine (CRE) and blood urea nitrogen (BUN) levels (Figure 2C-D) between the groups after 7 days. Mouse hemogram was also determined, and the results showed that Se@BSA NPs did not influence the red blood cell, white blood cell, and platelet counts or the hemoglobin content in mouse blood (Figure S5). No pathological changes were observed in vital organs, including the heart, liver, spleen, lungs, and kidneys (Figure 2E), which indicated the biocompatibility of Se@BSA NPs *in vivo*.

To investigate the biodistribution of Se@BSA NPs *in vivo*, mice were given 1 mg/kg Se@BSA NPs by intravenous injection. It was found that the NPs were almost eliminated from blood after 24 h (Figure S6). Fluorescence imaging showed that Se@BSA NPs, which were probed by fluorescein isothiocyanate (FITC), were primarily distributed in mouse kidneys and liver (Figure 2F), consistent with the results revealed by ICP-MS (Figure 2G). The kidneys maintained a high level of Se@BSA NPs, particularly 24 h after the initial injection, which was in accord with the accumulation of Ceria NPs in AKI mice 31. Due to endocytosis by renal tubular epithelial cells (Figure 2H and Figure S7), Se@BSA NPs mostly accumulated in the renal tubules, as FITC-labeled NPs were colocalized with aquaporin 1 (AQP1, Figure 2I), a recognized marker for renal proximal tubules 32. Since tubulointerstitial inflammation is a common characteristic of AKI 33, the distribution of Se@BSA NPs in renal tubules allowed a potential application in AKI treatment.

### Mitigation of IRI-AKI by Se@BSA NPs

**Figure 3:**
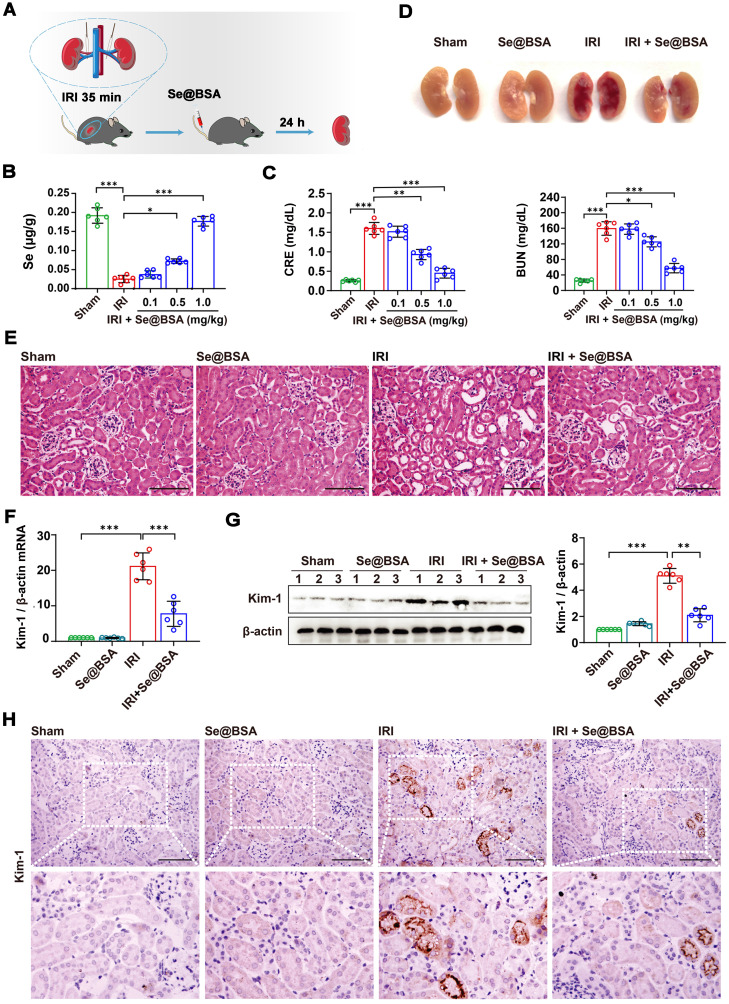
** (A)** Diagram depicting the establishment of the IRI-AKI mouse model. **(B)** Se contents in the kidneys of IRI-AKI mice. The results are shown as the mean ± SD. ^*^, *P* < 0.05; ^***^, *P* < 0.001. **(C)** CRE and BUN contents in IRI-AKI mice after Se@BSA NP treatment. The results are shown as the mean ± SD. ^*^, *P* < 0.05; ^**^, *P* < 0.01; ^***^, *P* < 0.001. **(D)** Pictures of mouse kidneys. **(E)** HE staining of the kidneys of IRI-AKI mice treated with Se@BSA NPs. The scale bar indicates 200 µm. **(F)** Kim-1 mRNA expression relative to β-actin expression in mouse kidneys. The results are shown as the mean ± SD. ^***^, *P* < 0.001. **(G)** Western blotting detecting the Kim-1 expression in the kidneys. β-actin was used as the reference. Histogram shows the results of gray analysis for the protein bands. The results are shown as the mean ± SD. ^**^, *P* < 0.01; ^***^, *P* < 0.001. **(H)** Immunohistochemical staining showing the Kim-1 expression in the kidneys. The scale bar indicates 200 µm.

By clamping the renal pedicles of C57 mice for 35 min as previously described 34, we established an IRI-induced AKI model to evaluate the therapeutic efficacy of Se@BSA NPs (0.1, 0.5, and 1 mg/kg, I.V.). The mice were sacrificed for analysis at 24 h after tail vein injection (Figure 3A). The Se content in the kidneys of IRI-AKI mice was dramatically decreased (Figure 3B). Consistently, IRI caused overt kidney injury, manifested by higher CRE and BUN levels in IRI-AKI mice than in healthy mice (*P* < 0.001, Figure 3C). Se@BSA NP treatment alleviated Se deficiency and injury in a dose-dependent manner. Pathologic analysis indicated that IRI caused a large area of renal congestion (Figure 3D) and tubule injury (Figure 3E and Figure S8), which were both relieved by Se@BSA NPs (1 mg/kg, I.V.). The mRNA expression of kidney injury molecule (Kim)-1 was also detected. The results showed that Kim-1 was highly expressed in IRI-AKI mice but its levels were dramatically decreased after Se@BSA NP treatment (*P* < 0.001, Figure 3F). Western blotting (Figure 3G) and immunohistochemistry (Figure 3H) congruously confirmed this conclusion at the protein level. It is plausible that Se@BSA NPs are equipped with dramatic capacities to mitigate IRI-AKI *in vivo*.

### Suppression of NLRP3 inflammasome activation by Se@BSA NPs

**Figure 4:**
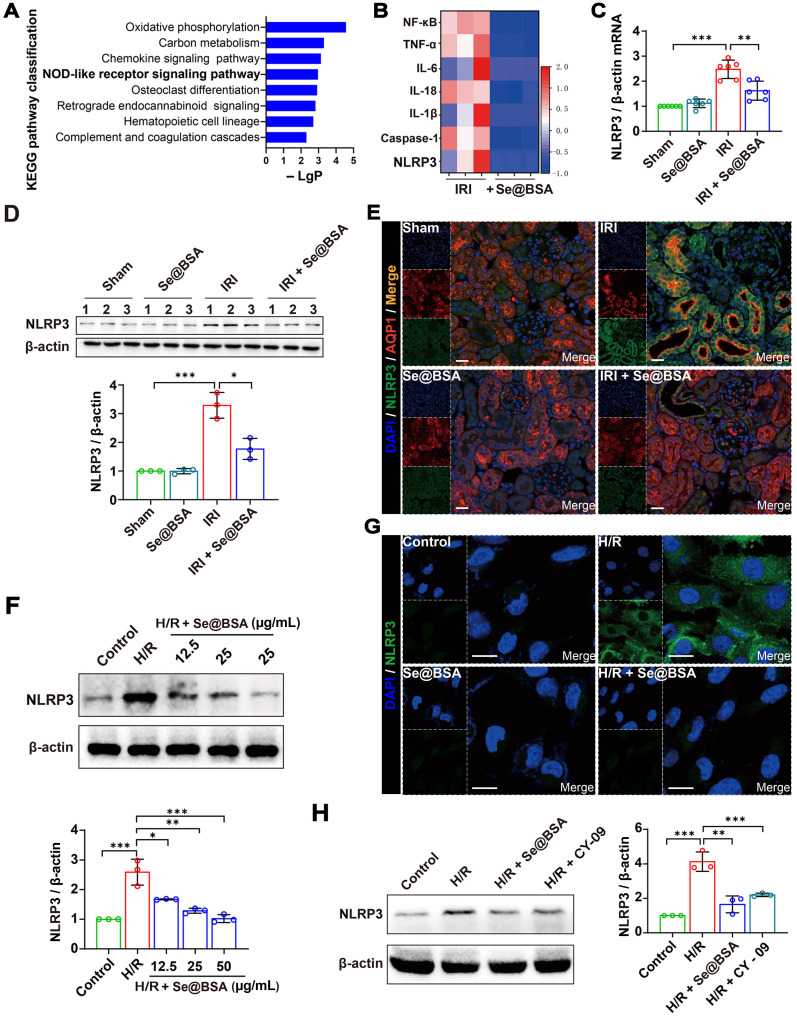
** (A)** Top eight enriched pathways in KEGG analysis. **(B)** Heat map showing the differential expression of NLRP3 inflammasome-related proteins in IRI-AKI mice treated with Se@BSA NPs. **(C)** NLRP3 mRNA expression relative to β-actin expression in mouse kidneys. The results are shown as the mean ± SD. ^**^, *P* < 0.01; ^***^, *P* < 0.001. **(D)** Western blotting detecting the NLRP3 expression in the kidneys. β-actin was used as the reference. Histogram shows the results of gray analysis for the protein bands. The results are shown as the mean ± SD. ^*^, *P* < 0.05; ^***^, *P* < 0.001. **(E)** Confocal images indicating the expression of NLRP3 (green) and AQP1 (red) in mouse kidneys. The scale bar indicates 20 µm. **(F)** Western blotting detecting the NLRP3 expression in HK-2 cells. β-actin was used as the reference. Histogram shows the results of gray analysis for the protein bands. The results are shown as the mean ± SD. ^*^, *P* < 0.05; ^**^, *P* < 0.01; ^***^, *P* < 0.001. **(G)** Confocal images indicating the expression of NLRP3 (green) in HK-2 cells. The scale bar indicates 20 µm. **(H)** Western blotting revealing the comparable inhibition of Se@BSA NPs to CY-09 on NLRP3 expression in HK-2 cells. β-actin was used as the reference. Histogram shows the results of gray analysis for the protein bands. The results are shown as the mean ± SD. ^**^, *P* < 0.01; ^***^, *P* < 0.001.

To gain insight into the underlying mechanisms of Se@BSA NPs in alleviating IRI-AKI, we performed a transcriptomic study by RNA-seq. A total of 4987 genes (2287 upregulated and 2700 downregulated) were altered in IRI-AKI mice after treatment with 1 mg/kg NPs, and the NOD-like receptor (NLR) signaling pathway was determined to be enriched *via* Kyoto Encyclopedia of Genes and Genomes (KEGG) analysis (Figure 4A). NLR thermal protein domain associated protein 3 (NLRP3), apoptosis-associated speck-like protein containing a CARD (ASC), and pro-cysteinyl aspartate specific proteinase-1 (pro-Caspase-1) constitute the NLRP3 inflammasome 35. Previous studies have shown that NLRP3 inflammasome activation contributes to renal dysfunction 36; conversely, inhibiting the activation of the NLRP3 inflammasome is beneficial for curbing contrast-induced AKI 37.

Our sequencing and q-PCR data showed that IRI induced a remarkable upregulation of NLRP3 levels (Figure 4B-C). The protein level was significantly higher in IRI-AKI mice than in healthy mice (*P* < 0.001), whereas this high protein level was visibly reduced after Se@BSA NP treatment (Figure 4D-E). Alternatively, HK-2 cells were subjected to hypoxia/reoxygenation (H/R) to mimic IRI-induced renal tubular injury *in vitro* 38. NLRP3 expression levels were increased in H/R cells (Figure 4F-G), and the addition of 50 μg/mL Se@BSA NPs almost eliminated NLRP3 accumulation in the cytoplasm after 6 h to an extent that was comparable to that achieved with the use of 10 μg/mL CY-09 inhibitor for suppressing NLRP3 inflammasome activation 39 (Figure 4H). Additionally, the reduction in NLRP3 levels in H/R cells induced by Se@BSA NPs occurred in a dose-dependent manner (Figure 4F and Figure S9).

**Figure 5:**
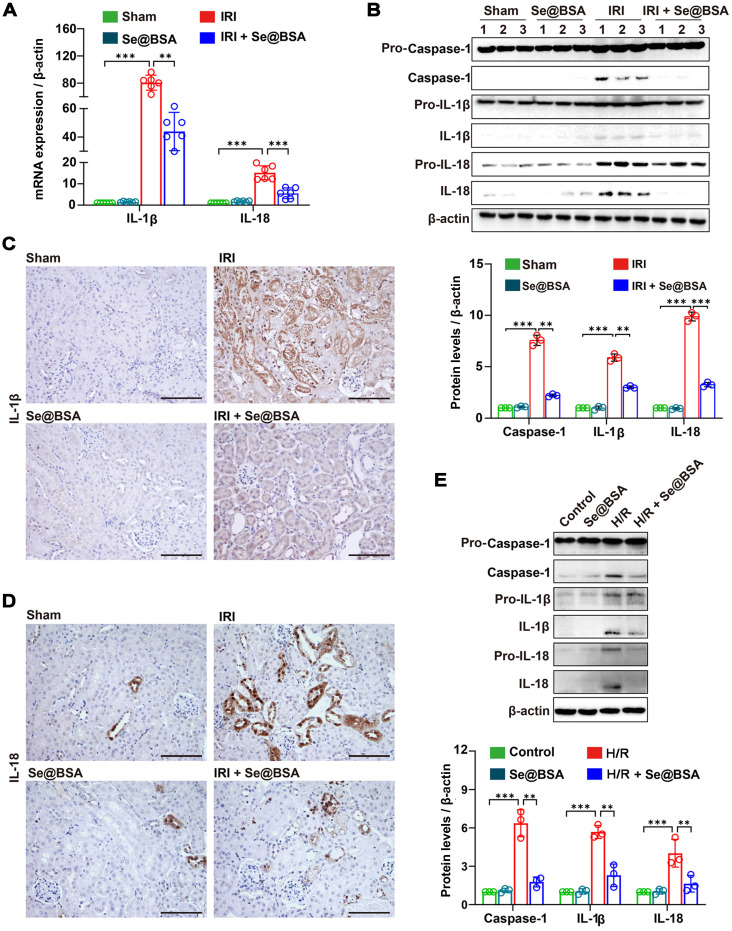
** (A)** IL-1β and IL-18 mRNA expression relative to β-actin in mouse kidneys. The results are shown as the mean ± SD. ^**^, *P* < 0.01; ^***^, *P* < 0.001. **(B)** Western blotting detecting the IL-1β, IL-18, and Caspase-1 expression in the kidneys. β-actin was used as the reference. Histogram shows the results of gray analysis for the protein bands. The results are shown as the mean ± SD. ^**^, *P* < 0.01; ^***^, *P* < 0.001. **(C)** Immunohistochemical staining showing the IL-1β and IL-18** (D)** expression in the kidneys. The scale bar indicates 200 µm. **(E)** Western blotting detecting the IL-1β, IL-18, and Caspase-1 expression in HK-2 cells. β-actin was used as the reference. The results of gray analysis are presented as the mean ± SD. ^**^, *P* < 0.01; ^***^, *P* < 0.001.

Assembly of the NLRP3 inflammasome enabled the proteolytic cleavage of pro-Caspase-1 into active Caspase-1, resulting in the maturation of the inflammatory factors IL-1β and IL-18 from the cytokine precursors pro-IL-1β and pro-IL-18, respectively 40. Guided by the sequencing results shown in Figure 4B, we determined the mRNA expression levels of IL-1β and IL-18 in the kidneys and discovered significant higher levels in IRI-AKI mice than in healthy mice (*P* < 0.001, Figure 5A). High levels of IL-1β, IL-18, and Caspase-1 (Figure 5B) were observed in the kidneys of IRI-AKI mice. However, after treatment with 50 μg/mL Se@BSA NPs, the levels of these proinflammatory factors were all reduced (Figure 5C-D). Similar findings were obtained in cell experiments in which levels of IL-1β, IL-18, and Caspase-1 expression in H/R cells were markedly decreased by Se@BSA NPs (*P* < 0.01, Figure 5E).

### Modulation of GPx-1 expression by Se@BSA NPs

**Figure 6:**
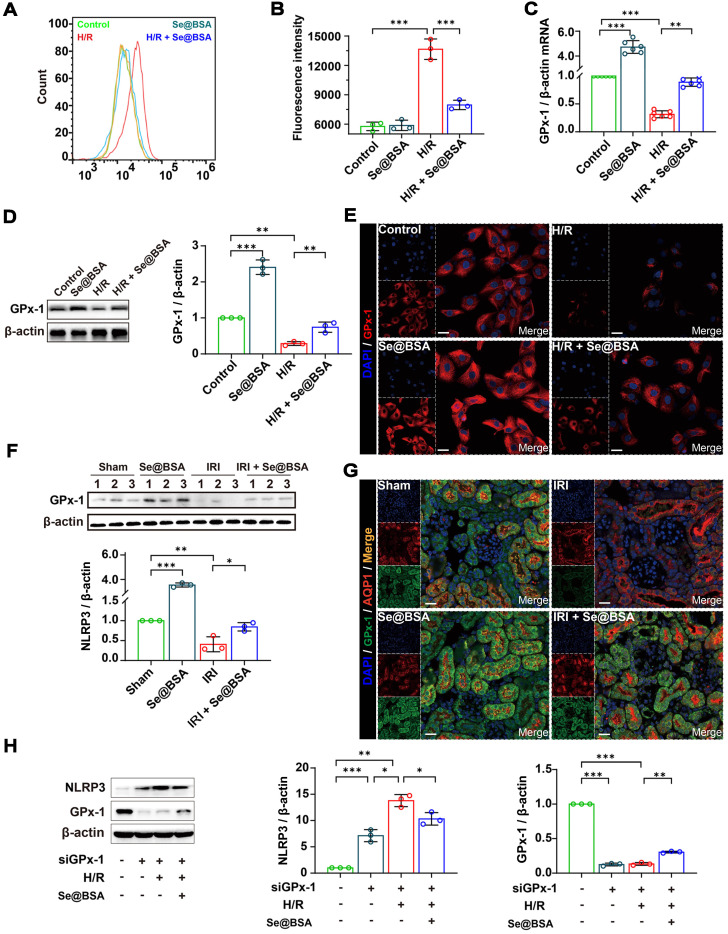
** (A)** Counts of fluorescent cells by flow cytometry. **(B)** Average fluorescence intensity of HK-2 cells. The results are shown as the mean ± SD. ^**^, *P* < 0.01; ^***^, *P* < 0.001. **(C)** GPx-1 mRNA expression relative to β-actin in HK-2 cells. The results are shown as the mean ± SD. ^**^, *P* < 0.01; ^***^, *P* < 0.001. **(D)** Western blotting detecting the GPx-1 expression in HK-2 cells. β-actin was used as the reference. The results of gray analysis are presented as the mean ± SD. ^**^, *P* < 0.01; ^***^, *P* < 0.001.** (E)** Confocal images indicating the expression of GPx-1 (red) in HK-2 cells. The scale bar indicates 20 µm. **(F)** Western blotting detecting the GPx-1 expression in mouse kidneys. β-actin was used as the reference. The results of gray analysis are shown as the mean ± SD. ^*^, *P* < 0.05; ^**^, *P* < 0.01; ^***^, *P* < 0.001. **(G)** Confocal images showing the expression of GPx-1 (green) and AQP1 (red) in mouse kidneys. The scale bar indicates 20 µm. **(H)** Western blotting detecting the NLRP3 and GPx-1 expression in HK-2 cells. The results of gray analysis are shown as the mean ± SD. ^*^, *P* < 0.05; ^**^, *P* < 0.01; ^***^, *P* < 0.001.

The NLRP3 inflammasome can be activated by ROS 3,41, which are excessively generated in various tissues after IRI 42. Our flow cytometry data supported that the ROS levels were elevated in HK-2 cells after H/R treatment (Figure 6A). Nevertheless, the average fluorescence intensity of 2',7'-dichlorodihydrofluorescein diacetate (DCFH-DA), an effective probe for ROS 43, in H/R cells was decreased from 13662.4 ± 1046.9 to 8303.9 ± 406.7 after coincubation with 50 μg/mL Se@BSA NPs (Figure 6B). Of note, the ROS reduction identified with an increase of GPx-1 (Figure 6C), one of the most abundant GPx isoforms in eukaryocytes 44.

Previous studies have shown that GPx-1-deficient mice were susceptible to oxidative stress and had abnormalities in the myocardial vasculature after IRI 45. Otherwise, overexpression of GPx-1 could mitigate IRI-induced tissue damage 46. We found an overt reduction in GPx-1 levels in HK-2 cells following H/R treatment in the present study (Figure 6D-E). Remarkably, an upregulation of GPx-1 levels was detected after Se@BSA NP treatment, and the modulation of GPx-1 expression levels by this nanomaterial manifested in a dose-dependent manner (Figure S10). Similar results were also found in IRI-AKI mice, in which the GPx-1 levels in the kidneys were markedly increased after Se@BSA NP treatment (1 mg/kg, I.V., Figure 6F-G). To decipher the connection between GPx-1 and NLRP3, we suppressed GPx-1 expression in HK-2 cells by siRNA. Levels of NLRP3 expression were found to be increased in normal cells upon GPx-1 reduction (Figure 6H), indicating that GPx-1 inhibits NLRP3 production. Due to the induced expression of GPx-1, the level of NLRP3 was significantly lower in H/R cells exposed to NPs than in cells without treatment. Collectively, these data demonstrated that GPx-1 modulation contributed to the suppression of NLRP3 inflammasome activation by Se@BSA NPs.

### Inhibition of renal fibrosis by Se@BSA NPs

**Figure 7:**
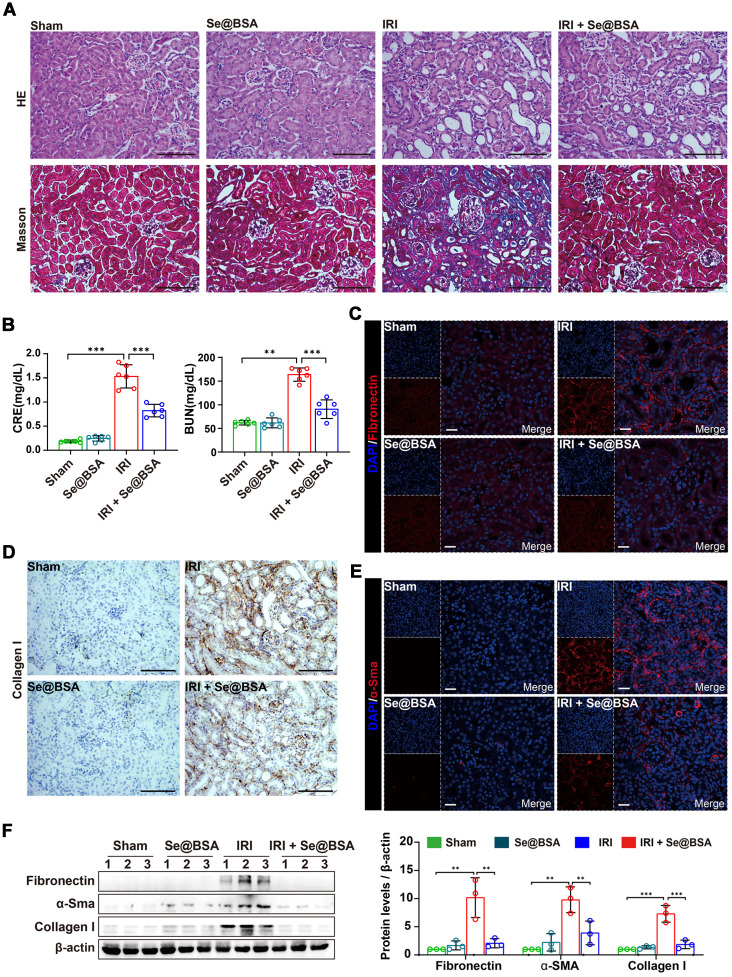
** (A)** HE and Masson staining of the kidneys of IRI-AKI mice in the absence and presence of Se@BSA NP treatment. The scale bar indicates 200 µm. **(B)** Determination of CRE and BUN contents in IRI-AKI mice at 2 weeks after Se@BSA NP treatment. The results are shown as the mean ± SD. ^**^, *P* < 0.01; ^***^, *P* < 0.001. **(C)** Confocal images showing the expression of fibronectin (red) and α-Sma (**E,** red) in mouse kidneys. The scale bar indicates 20 µm. **(D)** Immunohistochemical staining showing the collagen I expression in the kidneys. The scale bar indicates 200 µm. **(F)** Western blotting detecting the fibronectin, α-Sma and collagen I expression in mouse kidneys. The results of gray analysis are shown as the mean ± SD. ^**^, *P* < 0.01; ^***^, *P* < 0.001.

Maladaptive repair after AKI predisposes patients to the development of chronic kidney disease, which is primarily characterized by renal fibrosis 47. To investigate the influence of Se@BSA NP treatment on the outcomes of AKI, we performed a pathological study on IRI-AKI mice after 14 days. As shown in Figure 7A, severe tubular injuries and an expanded interstitial area were observed in the kidneys of AKI mice. Intravenous administration of 1 mg/kg Se@BSA NPs obviously attenuated kidney injury, resulting in improved renal function (Figure 7B). Markedly decreased expression levels of fibrosis indicators, including fibronectin (Figure 7C), collagen I (Figure 7D), and α-Sma (Figure 7E), were also found in IRI-AKI mice after Se@BSA NP treatment, consistent with the conclusion drawn by the results of Western blotting (Figure 7F), thus suggesting an inhibitory effect of the nanomaterial on fibrosis formation after AKI.

## Conclusions

Se is an essential trace element abundant in the kidney, and its levels decrease upon renal dysfunction. In this study, we fabricated a nanomaterial comprising Se^0^ and BSA using a simple method and applied it to treat IRI-induced AKI. The nanomaterial Se@BSA NPs upregulated GPx-1 levels and inhibited NLRP3 inflammasome activation in renal tubular epithelial cells stimulated with hypoxia/reoxygenation, resulting in reductions in the levels of active Caspase-1 and the proinflammatory factors IL-1β and IL-18. Se@BSA NPs maintained a high level in the kidneys after intravenous injection. Owing to modulation of the GPx-1/NLRP3/Caspase-1 pathway, Se@BSA NPs ameliorated the renal function of IRI-AKI mice in a dose-dependent manner and dramatically arrested the development of renal fibrosis after AKI. Considering the shortage of effective measures for AKI in the clinic, we believe that the biocompatible and easy-to-produce nanomaterial may be promising therapeutic candidates.

## Experimental Section

### Materials

BSA (A1933), ascorbic acid (PHR1008), and sodium selenate (S0882) were obtained from Sigma Aldrich (Shanghai, CHN). FITC-labeled BSA was purchased from Xi'an ruixi Biological Technology (R-FB-005). Paraformaldehyde (P0099), sodium citrate (P0081), hematoxylin eosin (HE) staining kit (C0105S), hematoxylin staining solution (C0107), and sodium dodecyl sulfate-polyacrylamide gel electrophoresis (SDS-PAGE) kit (P0012A) were purchased from Beyotime (Shanghai, CHN). Dialysis bags (8000-14,000 D) were purchased from Solarbio (YA1073, Beijing, CHN). Dulbecco's modified Eagle's medium (DMEM, 11995065) and fetal bovine serum (FBS, 10100147) were obtained from Gibco (Thermo Fisher Scientific, Shanghai, CHN). Cell Counting Kit-8 (CCK-8) was purchased from Dojindo (Shanghai, CHN).

### Preparation of Se@BSA NPs

Forty milligrams of BSA dissolved in 10 mL sterile water was coincubated with 8 mL ascorbic acid (50 mM) at room temperature for 0.5 h. Sodium selenate (50 mM, 4 mL) was added, and the complex was incubated for 12 h with agitation at 1000 rpm. Se@BSA NPs collected by dialysis at room temperature for 24 h were freeze-dried for evaluation 48. FITC-labeled NPs were fabricated using the same method, in which BSA probed with FITC was employed.

### Characterization of Se@BSA NPs

Se@BSA NPs were visualized by transmission electron microscopy (TEM, JEM-1400 PLUS, JEOL, Shanghai, CHN). The elemental mapping of the nanomaterial was investigated using field emission TEM (Talos F200S, FEI, Hillsboro, OR, US) coupled with EDS analysis. XPS was carried out with an ESCALAB 250Xi spectrometer (Thermo Fisher Scientific) equipped with an achromatic Al-Kα X-ray source. FT-IR (Nicolet iS5, Thermo Fisher Scientific) was performed to determine the infrared absorption spectrum of BSA in Se@BSA NPs. The particle size and ζ-potential of Se@BSA NPs were determined with a Nano-ZS instrument (Malvern, Worcestershire, UK) at room temperature.

### Toxicological evaluation

The cytotoxicity of Se@BSA NPs was evaluated using HK-2 cells and erythrocytes. HK-2 cells obtained from the cell bank of the Chinese Academy of Sciences (CAS, Shanghai, CHN) were cultured with DMEM containing 10% FBS and seeded into a 96-well plate at a density of 5 × 10^3^ cells per well. The survival of cells exposed to increasing concentrations of Se@BSA NPs (12.5, 25, 50, 100, and 200 μg/mL, based on the weight of NP) was detected by CCK-8 after 24 h. A hemolytic experiment was conducted as we recently described 49. Briefly, a total of 300 μL mouse erythrocytes diluted in 0.9% NaCl solution was incubated with 1.2 mL Se@BSA NPs at 37 °C for 2 h. The absorbance of the supernatant at 405 nm was detected using a microplate reader. These experiments were conducted in triplicate and repeated twice on different days.

The *in vivo* toxicity of Se@BSA NPs was evaluated by mouse experiments. Mice were cared for and treated in accordance with the NIH guidelines for the care and use of laboratory animals (NIH Publication no. 85e23 Rev. 1985). Twelve male eight-week-old C57 mice were randomly divided into two groups (n = 6). Se@BSA NPs (20 mg/kg) were administered by intravenous injection. Mouse blood obtained at day 7 after injection was analyzed on a Sysmex XT-2000i fully automatic hematology analyzer (Kobe, JPN). BUN and CRE, two renal function indicators, were measured using a urea assay kit (C013-2-1, Nanjing Jiancheng Bioengineering Institute, Jiangsu Province, CHN) and a creatinine assay kit (C011-2-1, Nanjing Jiancheng Bioengineering Institute), respectively. Meanwhile, the vital organs were obtained and sectioned for HE staining. An Olympus DX51 optical microscope (Tokyo, Japan) was employed to observe the pathological changes. All mouse experiments in this study were approved by the Animal Experimental Ethics Committee of TMMU (AMUWEC20211133).

### Biodistribution analysis

Thirty male eight-week-old C57 mice were randomly divided into five groups (n = 6). The FITC-labeled NPs were intravenously administered at a dose of 1 mg/kg. The mice were sacrificed at 0, 3, 6, 12, and 24 h post injection to obtain vital organs, including the heart, liver, spleen, lungs, and kidneys. The distribution of Se@BSA NPs in these organs was visualized with an IVIS Spectrum Imaging System (PerkinElmer, Shanghai, CHN). ICP-MS (Agilent, Santa Clara, CA, USA) was applied to determine the Se content in the organs. The accumulation of FITC-labeled NPs in the kidney tubules at 24 h post-initial injection was analyzed by immunofluorescence.

### Cell experiments

HK-2 cells were seeded into a 12-well plate with sterile glass slides at a density of 1 × 10^5^ cells per well and cultured in DMEM containing 10% FBS. After adherence, the cells were exposed to 50 μg/mL FITC-labeled Se@BSA NPs at 37 °C for 6 h. The endocytosis of NPs was observed by a Zeiss LSM 780 NLO confocal microscope. To induce H/R injury, HK-2 cells were seeded into 6-well plates at a density of 2 × 10^5^ cells per well and initially cultured in a regular incubator (5% CO_2_ and 95% air) for 12 h. The culture medium was then removed, and Hank's balanced salt solution (HBSS) was added. The cells were transferred to a hypoxic incubator (94% N_2_, 5% CO_2_ and 1% O_2_) and cultured for 24 h. After removal of HBSS, regular medium and Se@BSA NPs (12, 25, and 50 μg/mL) were subsequently added. Reoxygenation was performed by culturing the cells in a regular incubator for 6 h. The cells were then harvested and processed for biochemical analysis. GPx-1 expression in HK-2 cells was suppressed by a 21-nt siRNA (sense, AUUCAGAAUCUCUUCGUUCUU; antisense, GAACGAAGAGAUUCUGAAUUC) using the Lipofectamine 3000 transfection reagent (L3000-015, Invitrogen, Thermo Fisher Scientific).

### IRI-AKI mouse experiment

Fifty-four male eight-week-old C57 mice were randomly divided into nine groups (n = 6). IRI-AKI was induced by clamping the renal pedicles for 35 min as previously described 50. Se@BSA NPs (0.1, 0.5, and 1 mg/kg) were given by intravenous injection after reperfusion. The mice in the sham group received operations in which the kidneys were exposed but not clamped, and they were treated with sterile PBS. After reperfusion for 24 h, the mice were sacrificed, and the blood and kidneys were obtained for analysis. Tubular injury scores were calculated according to the percentage of damaged tubules: 0, no damage; 1, < 25%; 2, 25 to 50%; 3, 50 to 75%, 4, > 75% 51. At day 14 after IRI-AKI, renal fibrosis was investigated by detecting fibronectin, collagen I and α-Sma levels. The kidney sections were analyzed by Masson staining using a Nanjing Jiancheng Bioengineering Institute kit (D026-1).

### RNA-seq

Total RNA was extracted from the kidneys of IRI-AKI mice in the absence and presence of Se@BSA NP treatment (1 mg/kg, I.V.) using a TaKaRa RNAiso Plus reagent. Library construction and RNA sequencing were conducted by Tsingke Biotechnology (Beijing, CHN). Briefly, mRNA was purified by oligo(dT)-attached magnetic beads. Random hexamer-primed reverse transcription and second-strand cDNA synthesis were applied to obtain cDNA. The final library was amplified to make DNA nanoballs (DNBs), which were loaded into the patterned nanoarray. Sequencing was performed using the BGISEQ-500 platform, and the data were filtered with SOAPnuke (version 1.5.2) 52. The expression level of the gene was calculated by StringTie (version 2.1.2) 53. Differential expression analysis was performed using DESeq2 (version 1.4.5) 54 with a Q value ≤ 0.05. KEGG analysis was performed by Phyper (https://en.wikipedia.org/wiki/Hypergeometric_distribution).

### Immunofluorescence and immunohistochemistry staining

HK-2 cells and mouse kidney sections were fixed with 4% paraformaldehyde for 15 min and blocked with 1% BSA for 1 h. The antibodies used in immunofluorescence staining are shown in Table S1. A Zeiss LSM 780 NLO confocal microscope was applied to observe the cells and tissues. To perform the immunohistochemistry staining, the tissues were fixed in 4% paraformaldehyde, embedded in paraffin, and sectioned at 3 µm. The tissue sections were deparaffinized and incubated with 0.1 M sodium citrate (pH 6.0) at 95 °C for 30 min. The antibodies used in staining are shown in Table S2. A 3,3'-diaminobenzidine (DAB) substrate kit (ab64238, Abcam) was applied for visualization.

### q-PCR

RNA from mouse kidneys and HK-2 cells was extracted with TaKaRa (Dalian, Liaoning, CHN) RNAiso Plus reagent and reverse transcribed using a PrimeScript RT-PCR kit (DRR014A, TaKaRa). The primer sequences are presented in Table S3. The data were processed with a Bio-Rad iQ5 standard-edition optical system (version 2.1). This assay was performed in duplicate and repeated three times on different days.

### Western blotting

Cells and tissues were collected and processed for protein extraction. A total of 25 μg of each sample was resolved by 10% SDS-PAGE. Afterward, the proteins were transferred onto a 0.22 µm polyvinylidene difluoride membrane (PVDF, Millipore, USA). The antibodies used in immunoblotting are shown in Table S4. A BeyoECL Plus chemiluminescence kit (P0018S, Beyotime) was applied to observe the protein bands. Grayscale analysis was performed with ImageJ (version 1.8.0). The experiments were repeated three times on different days.

### Statistical analysis

Statistical analysis was performed with GraphPad Prism 8 (GraphPad Software Inc, La Jolla, CA, USA) using the LSD multiple-comparison test and two-tailed paired t test. *P* < 0.05 was considered to be statistically significant.

## Supplementary Material

Supplementary figures and tables.

Supplementary sequencing data.

## Abbreviations

AKI: acute kidney injury
IRI: ischemia reperfusion injury
Se: selenium
BSA: bovine serum albumin
NP: nanoparticle
H/R: hypoxia/reoxygenation
GPx-1: glutathione peroxidase-1
NLRP3: NOD-like receptor thermal protein domain associated protein 3
Caspase: cysteinyl aspartate specific proteinase
ROS: reactive oxygen species
SIRS: systemic inflammatory response syndrome
EDS: energy dispersive X-ray spectroscopy
XPS: X-ray photoelectron spectroscopy
FT-IR: Fourier transform-infrared spectrometry
DLS: dynamic light scattering
CRE: creatinine
BUN: blood urea nitrogen
I.V.: intravenous injection
FITC: fluorescein isothiocyanate
ICP-MS: inductively coupled plasma-mass spectrometry
AQP1: aquaporin 1
Kim-1: kidney injury molecule-1
KEGG: Kyoto Encyclopedia of Genes and Genomes
PCR: polymerase chain reaction
IL: interleukin
DCFH-DA: 2',7'-dichlorodihydrofluorescein diacetate
TEM: transmission electron microscopy
SDS-PAGE: sodium dodecyl sulfate-polyacrylamide gel electrophoresis
DMEM: Dulbecco's modified Eagle's medium
FBS: fetal bovine serum
HE: hematoxylin eosin
DAPI: 2-(4-amidinophenyl)-6-indolecarbamidine
