# Supplementary material for: Selenium nanoparticles alleviate ischemia reperfusion injury-induced acute kidney injury by modulating GPx-1/NLRP3/Caspase-1 pathway
Source: Theranostics. 2022 May 9;12(8):3882–95. doi: 10.7150/thno.70830 (PMC9131270; doi:10.7150/thno.70830)

Supplementary Material

Selenium nanoparticles alleviate ischemia reperfusion injury-induced acute kidney injury by modulating GPx-1/NLRP3/Caspase-1 pathway

Shaobo Wang, Yin Chen, Songling Han, Yong Liu, Jining Gao, Yinghui Huang, Wei Sun, Junping Wang, Cheng Wang, Jinghong Zhao

Corresponding authors: E-mail: zhaojh@tmmu.edu.cn, wangctmmu@126.com, wangjunping@tmmu.edu.cn

Table of contents:

Table S1. Antibodies applied in the immunofluorescence staining

Table S2. Antibodies applied in the immunohistochemistry staining

Table S3. Sequences of primers used in q-PCR

Table S4. Antibodies applied in the Western blotting

Figure S1. SDS-PAGE determining the existence of BSA in the NPs

Figure S2. Zeta potential and particle size of different batches of Se@BSA NPs

Figure S3. Changes in body weight at day 7 post Se@BSA NP treatment

Figure S4. Changes in ALT and AST levels at day 7 post Se@BSA NP treatment

Figure S5. Changes in mouse hemogram at day 7 post Se@BSA NP treatment

Figure S6. ICP-MS detecting the Se contents in serum post Se@BSA NP treatment

Figure S7. ICP-MS detecting the Se contents in HK-2 cells post Se@BSA NP treatment

Figure S8. Tubular injury score of IRI-AKI mouse

Figure S9. NLRP3 mRNA expression in HK-2 cells

Figure S10. Induced expression of GPx-1 in HK-2 cells by Se@BSA NPs

Table S1. Antibodies applied in the immunofluorescence staining

**Proteins**	**Primary Antibodies**	**Secondary Antibodies**
Mouse AQP1	1:100, sc-25287 (Santa Cruz, Shanghai, CHN)	Cy3, A0521 (Beyotime)
NLRP3	1:200, bs-23722R (Bioss, Beijing, CHN)	Alexa Fluor 488, A0423 (Beyotime)
GPx-1	1:100, AF7017 (Beyotime)	Cy3, A0516 (Beyotime)
Fibronectin	1:100, ab2413 (Abcam, Shanghai, CHN)	Cy3, A0516 (Beyotime)
α-Sma	1:200, 19245 (CST, Shanghai, CHN)	Cy3, A0516 (Beyotime)

Table S2. Antibodies applied in the immunohistochemistry staining

Proteins	Primary Antibodies	Secondary Antibodies
Kim-1	1:100, ab78494 (Abcam)	ZLI-9019 (ZSGB-BIO, Beijing, CHN)
IL-1β	1:100, ab283818 (Abcam)	ZLI-9019 (ZSGB-BIO)
IL-18	1:100, ab191860 (Abcam)	ZLI-9019 (ZSGB-BIO)
Collagen I	1:1000, 81375 (CST)	ZLI-9019 (ZSGB-BIO)

Table S3. Sequences of primers used in q-PCR

Species	Name	Sequence (5'-3')		Product length
Mouse	Kim-1	Forward	CAGGGTCTCCTTCACAGCAG	172
		Reverse	CCACCACCCCCTTTACTTCC	
	GPx-1	Forward	AATGTCGCGTCTCTCTGAGG	117
		Reverse	TCCGAACTGATTGCACGGG	
	NLRP3	Forward	ATCAACAGGCGAGACCTCTG	96
		Reverse	GTCCTCCTGGCATACCATAGA	
	IL-1β	Forward	TTCAGGCAGGCAGTATCACTC	166
		Reverse	CCAGCAGGTTATCATCATCA	
	IL-18	Forward	CCAGACCAGACTGATAATATAC	110
		Reverse	TCTTGTTCTTACAGGAGAGG	
	β-actin	Forward	CACTGTCGAGTCGCGTCC	89
		Reverse	TCATCCATGGCGAACTGGTG	
Human	Kim-1	Forward	CTTCTAACTAACCGGCTGATCC	244
		Reverse	TTCCGATAGGTGACGTGGGT	
	GPx-1	Forward	CAGTCGGTGTATGCCTTCTCG	105
		Reverse	GAGGGACGCCACATTCTCG	
	NLRP3	Forward	GATCTTCGCTGCGATCAACAG	81
		Reverse	CGTGCATTATCTGAACCCCAC	
	IL-1β	Forward	AGCCATGGCAGAAGTACCTG	116
		Reverse	CCTGGAAGGAGCACTTCATCT	
	IL-18	Forward	ATCGCTTCCTCTCGCAACAA	223
		Reverse	GAGGCCGATTTCCTTGGTCA	
	β-actin	Forward	GATTCCTATGTGGGCGACGA	229
		Reverse	AGGTCTCAAACATGATCTGGGT	

Table S4. Antibodies applied in the Western blotting

Proteins	Primary Antibodies	Secondary Antibodies
β-actin	1:1000, AF50017 (Beyotime)	A0216 (Beyotime)
Kim-1	1:1000, ab78494 (Abcam)	A0208 (Beyotime)
NLRP3	1:1000, bs-23722R (Bioss)	A0208 (Beyotime)
IL-1β	1:1000, ab283818 (Abcam)	A0208 (Beyotime)
Mouse IL-18	1:1000, ab191860 (Abcam)	A0208 (Beyotime)
Human IL-18	1:1000, ab243295 (Abcam)	A0208 (Beyotime)
Caspase-1	1:1000, ab138483 (Abcam)	A0208 (Beyotime)
GPx-1	1:1000, AF7017 (Beyotime)	A0208 (Beyotime)
Fibronectin	1:1000, ab2413 (Abcam)	A0208 (Beyotime)
α-Sma	1:1000, 19245 (CST)	A0208 (Beyotime)
Collagen I	1:1000, 81375 (CST)	A0208 (Beyotime)

Figure S1. SDS-PAGE determining the existence of BSA in the NPs. A total of 200 μg of Se@BSA NPs was dissolved in 25 μL PBS. After a high speed centrifugation at 12 000 g for 15 min, the supernatant was collected. The precipitate was further dissolved in 25 μL PBS and heated for denaturation. Proteins were resolved by 10% SDS-PAGE and stained with Coomassie brilliant blue. BSA (5 μg) was employed as the positive control.

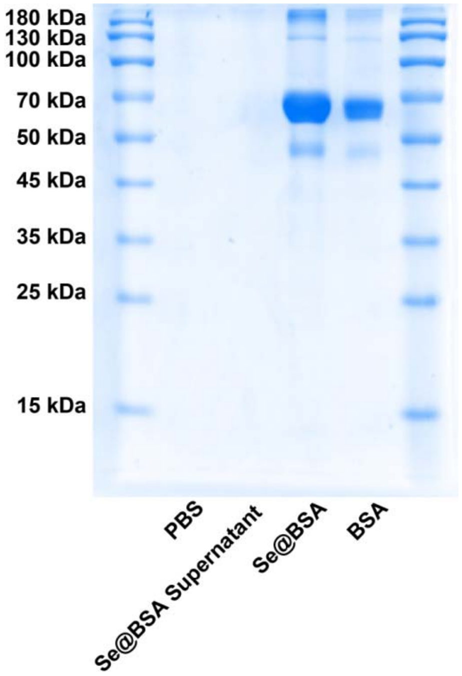

Figure S2. Zeta potential and particle size of different batches of Se@BSA NPs. (A) Zeta potential determination. (B) DLS detection of the hydrodynamic diameters of Se@BSA NPs. The results are presented as the mean ± SD.

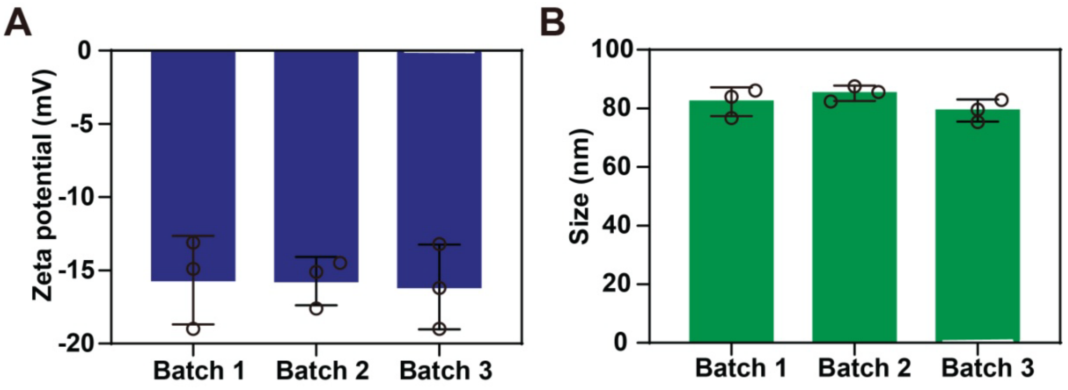

Figure S3. Changes in body weight at day 7 post Se@BSA NP treatment. Intravenous injection of 20 mg/kg Se@BSA NPs was innocuous to C57 mice. Mice in the sham group were treated with sterile PBS. n.s., not significantly.

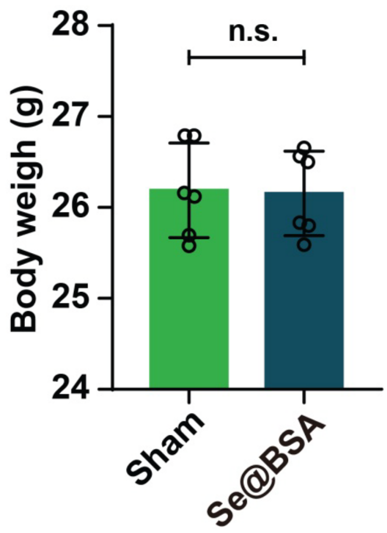

Figure S4. Changes in ALT and AST levels at day 7 post Se@BSA NP treatment. ALT and AST contents in mouse blood were determined by ELISA (SEA207Mu and SEB214Mu, CLOUD-CLONE CORP., Katy, TX, USA). n.s., not significantly.

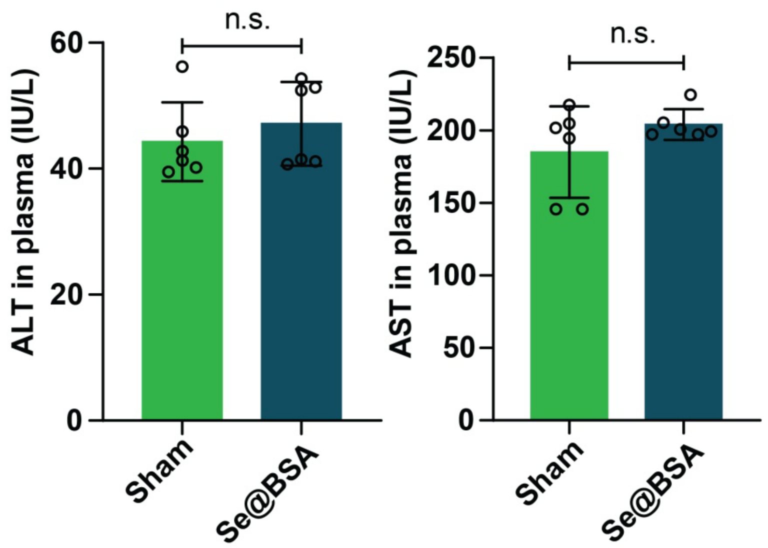

Figure S5. Changes in mouse hemogram at day 7 post Se@BSA NP treatment. Mouse blood was obtained at day 7 post Se@BSA NP (20 mg/kg, I.V.) treatment. Shown are the changes of (A) red blood cells, (B) white blood cells, (C) platelets, and (D) hemoglobin. n.s., not significantly.

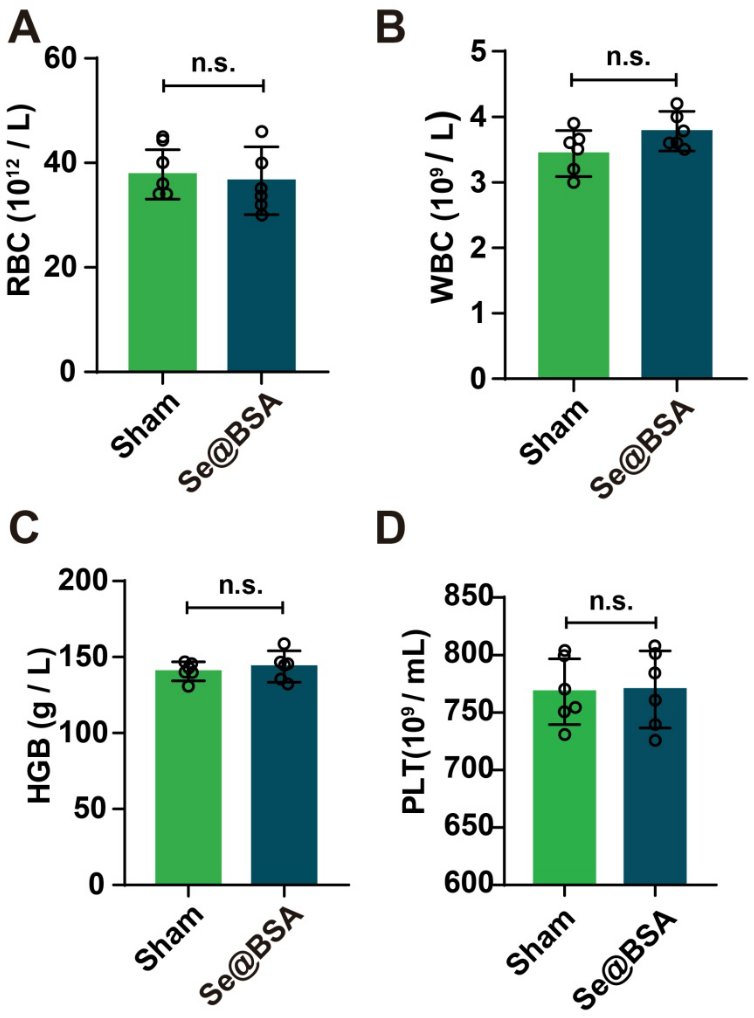

Figure S6. ICP-MS detecting the Se contents in serum post Se@BSA NP treatment. Mouse blood (20 μL) was obtained at 3, 6, 12, and 24 h post Se@BSA NP (1 mg/kg, I.V.) treatment. Samples were processed with 0.5 mL nitrohydrochloric acid for 24 h and adjusted to 1 mL, in which the Se contents were determined by ICP-MS. The results are presented as the mean ± SD.

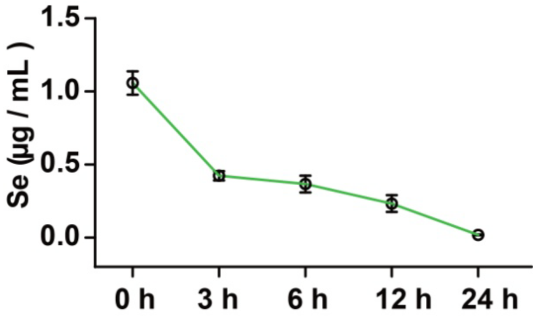

Figure S7. ICP-MS detecting the Se contents in HK-2 cells post Se@BSA NP treatment. Se@BSA NPs (50 μg/mL) were coincubated with 2×10^8 CFU HK-2 cells at 37 °C for 6 h. The cells were collected and processed with 2 mL nitrohydrochloric acid for 24 h. After dilution to 5 mL with 2% salpeter solution, the Se contents in the samples were determined by ICP-MS. The results are presented as the mean ± SD. ***, $P < 0.001$.

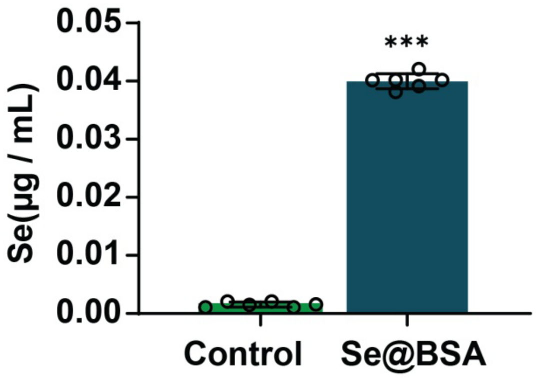

Figure S8. Tubular injury score of IRI-AKI mouse. The results are shown as the mean ± SD. ***, $P < 0.001$.

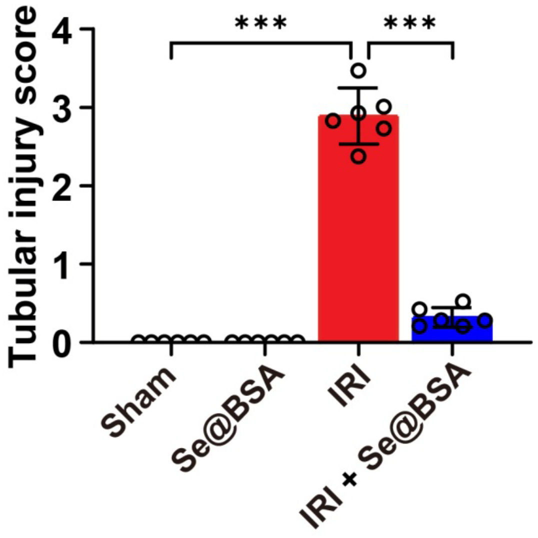

Figure S9. NLRP3 mRNA expression in HK-2 cells. H/R cells were exposed to different concentrations of NPs for 6 h. β-actin was used as the reference. The results are shown as the mean ± SD. *, $P < 0.05$; ***, $P < 0.001$.

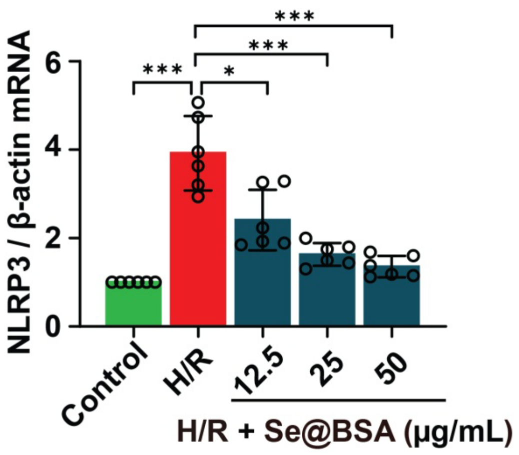

Figure S10. Induced expression of GPx-1 in HK-2 cells by Se@BSA NPs. (A) q-PCR detecting the mRNA expression of GPx-1. The results are shown as the mean ± SD. *, $P < 0.05$; **, $P < 0.01$; ***, $P < 0.001$. (B) Western blotting revealing the dose-dependent modulation of Se@BSA NPs on GPx-1 expression in HK-2 cells. β-actin was used as the reference.

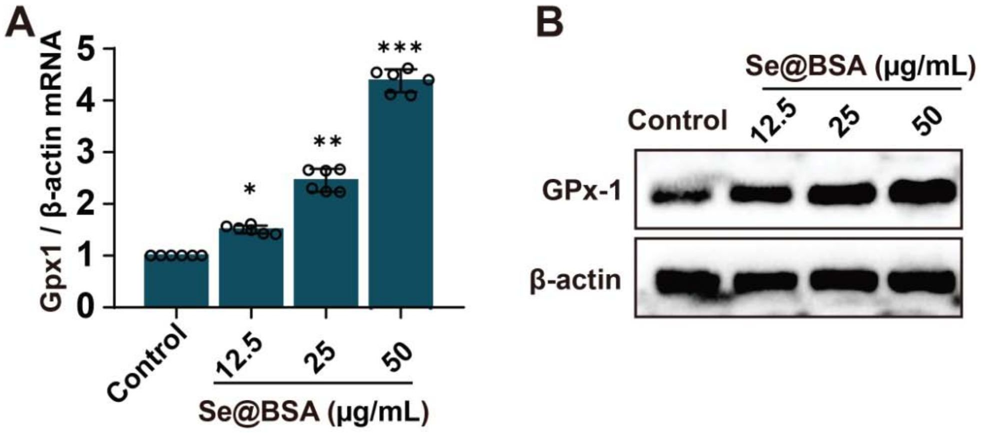

Supplement: Supplementary file 1 — Supplementary figures and tables. [file thnov12p3882s1.pdf]
